# Procalcitonin as a prognostic marker of patients with acute ischemic stroke

**DOI:** 10.1002/jcla.23301

**Published:** 2020-03-20

**Authors:** Ling Yan, Shuling Wang, Lanlan Xu, Zhen Zhang, Pu Liao

**Affiliations:** ^1^ Department of Clinical Laboratory Chongqing General Hospital Chongqing China

## Abstract

**Background:**

The information on mortality after an acute stroke patient is still limited.

**Objectives:**

The aim of this study was to investigate the prognostic potential of procalcitonin (PCT) serum levels in acute ischemic stroke.

**Methods:**

A total of 748 patients were enrolled in this study. Prognostic potential of PCT was evaluated by Kaplan‐Meier analysis and multivariable Cox hazard regression analyses.

**Results:**

Procalcitonin levels were found to be significantly higher in acute ischemic stroke patients who died in 30 days than those who survived. Univariate logistic regression analysis showed that PCT was significantly associated with 30‐day mortality, and Cox regression analysis revealed that PCT was a strong predictor of 30‐day overall mortality. Kaplan‐Meier analysis revealed that overall cumulative 30‐day mortality was significantly higher in those with PCT levels >1.5 ng/mL when compared to those with PCT levels <1.5 ng/mL.

**Conclusions:**

Procalcitonin is a significant independent prognostic marker of 30‐day mortality after the one set of acute ischemic stroke.

## 1 INTRODUCTION

Ischemic stroke is among the leading cause of mortality and remains serious long‐term disability worldwide. There were 2.5 million new stroke cases each year in China,^1^ and the incidence of stroke is predicted to rise because of the rapidly aging population. Although subsequent mortality has declined in recent years,^2^ a greater understanding of stroke biomarker of subsequent mortality will be required to establish appropriate prevention and treatment strategies.

Systematic inflammation plays a crucial role in the pathogenesis of ischemic stroke and may lead a secondary injury.^3^ Many inflammatory biomarkers, such as procalcitonin (PCT) and C‐reactive protein (CRP) are also identified as biomarkers of acute ischemic stroke.^4^, ^5^ PCT, a prohormone of calcitonin, is produced by C‐cells of the thyroid gland. In previous studies, PCT seemed to be a better marker for the diagnosis of infection than other biomarkers.^6^, ^7^, ^8^ Recent studies showed it may be a prognosis marker of ischemic stroke and may be better than CRP.^9^, ^10^ The concentration of PCT was transient increased after severe trauma and seemed proportional to the severity of tissue injury and hypovolemia.^11^ However, the relationship between PCT and short‐term outcome has not been well examined. In this study, we sought to determine the relationship between PCT and 30‐day all‐cause mortality after acute ischemic stroke in Chinese patients.

## 2 MATERIALS AND METHODS

We retrospective study all patients, less than 72 hours after symptom onset, with acute ischemic stroke event from January 2014 to December 2018 in a tertiary hospital. Acute ischemic stroke defined according to the World Health Organization criteria.^12^ We excluded patients with intracerebral hemorrhage, subarachnoid hemorrhage, systemic infections, transient ischemic attack, inflammation, and incomplete data.

For PCT measurement, the blood sample was collected by venipuncture within 48 hours after hospital admission. Blood samples were centrifuged at 2264 *g* for 10 minutes. The serum was measured within 2 hours after sample collected. PCT was measured in serum sample (Cobas e601, Roche). All‐cause of mortality defined as death occurring after hospital admission. All clinical data were collected from electronic medical records.

All data analyzed using SPSS 22.0. Two groups of any continuous variables were compared using Student's t test. Categorical variables were compared between groups using the chi‐square test. Survival analysis by multivariable Cox regression analysis was used to examine the risk factors for 30‐day mortality. The survival distribution function was estimated with the Kaplan‐Meier method, and a non‐parametric log‐rank test was used to compare the survival curves among the different groups. *P* < .05 was considered statistically significant.

## 3 RESULTS

A total of 748 patients were enrolled in this study. The median age was 68 years, with age ranged between 46 and 101. Demographic data, risk factors, laboratory finding, and 30‐day outcome were shown in Table 1. The inflammatory biomarkers including PCT, CRP, and neutrophils percentage in patients who died in 30 days were significantly higher than those who survived. Furthermore, the serum levels of PCT were also higher in patients with unfavorable functional outcome than those in patients with favorable functional outcome 1.37 ± 1.81 ng/mL vs 0.12 ± 0.41 ng/mL.

**Table 1:** Baseline characteristics of stroke patients

Characteristic	Death	Alive	*P* value
Median age (±SD)	75.19 ± 14.27	68.81 ± 13.73	.572
Male Sex	7 (24.14%)	307 (42.70%)	.047
Comorbidities			
Hypertension	21 (72.41%)	492 (68.43%)	.650
Coronary heart disease	7 (24.14%)	111 (15.44%)	.208
Laboratory finding			
PCT ng/mL	2.03 ± 2.57	0.14 ± 0.32	<.001
CRP mg/L	31.75 ± 69.81	22.76 ± 25.51	.022
White blood cell	3.53 ± 4.69	3.64 ± 3.50	.065
Neutrophile granulocyte %	85.55 ± 13.70	66.21 ± 10.63	<.001
Total bilirubin μmol/L	19.38 ± 11.99	12.7 ± 5.65	.019
Triglycerides mmol/L	1.02 ± 0.37	1.53 ± 1.45	.118
Urea mmol/L	8.35 ± 5.07	5.57 ± 2.13	.012

Abbreviations:CRP, C‐reactive protein, PCT, procalcitonin.

Death by the 30‐day follow‐up was associated with total bilirubin, urea, neutrophil percentage, and CRP (Table 2). Hypertension, coronary heart disease, total bilirubin >17 μmol/L, and urea >7.0 mmol/L were not associated with the mortality.

**Table 2:** Univariate logistic regression analysis of risk factors for mortality

Variable	HR	95% CI	*P* value
Sex	2.308	0.986‐5.403	.054
Hypertension	1.204	0.533‐2.717	.656
Coronary heart disease	1.741	0.744‐4.076	.201
CRP >5 mg/L	3.289	1.587‐6.813	.001
PCT >1.0 ng/mL	2.445	1.168‐5.119	.018
PCT >1.5 ng/mL	7.963	3.802‐16.678	<.001
Neutrophile granulocyte >70%	4.438	1.956‐10.020	<.001
Total bilirubin >17 μmol/L	1.190	0.573‐2.474	.641
Urea >7.0 mmol/L	2.091	0.926‐4.720	.076

Abbreviations:CRP, C‐reactive protein, PCT, procalcitonin.

**Figure 1:**
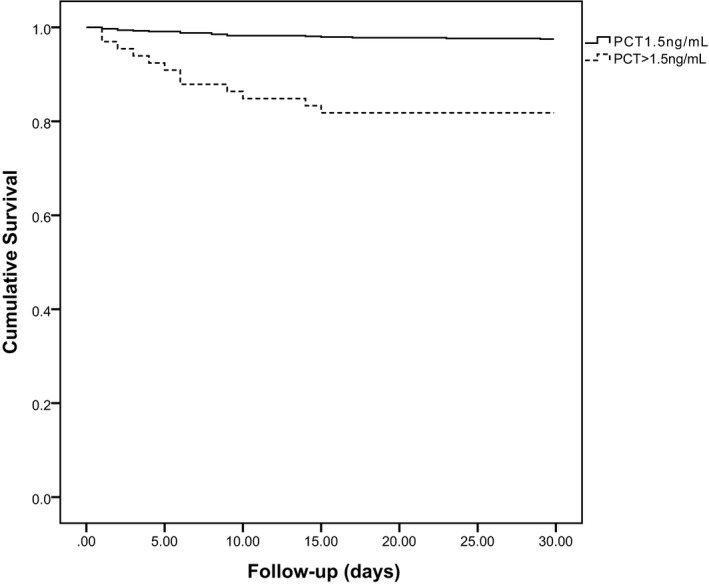
Kaplan‐Meier survival curves according to procalcitonin (PCT) at admission (*P* < .001)

Multivariate Cox regression analysis was used to analyze the independent risk factors for 30‐day mortality. As shown in Table 3, the inflammatory markers, PCT, CRP, and neutrophil percentage, were significant predictors of 30‐day overall mortality, with HR of 7.963, 3.289, and 4.438, respectively. Moreover, Kaplan‐Meier survival curves showed a trend for higher mortality of patients with PCT >1.5 ng/mL (Figure 1) (*P* < .001).

**Table 3:** Independent risk factors for mortality in patients with ischemic stroke

Variable	HR (95%CI)	*P* value
CRP >5 mg/L	2.242 (1.043‐4820)	.039
PCT >1.0 ng/mL	1.653 (0.750‐3.643)	.212
PCT >1.5 ng/mL	5.486 (2.508‐12.000)	<.001
Neutrophile granulocyte >70%	3.871 (1.707‐8.779)	.001

Abbreviations:CRP, C‐reactive protein, PCT, procalcitonin.

## 4 DISCUSSION

Stroke is the first leading cause of disability and over two‐thirds of stroke deaths worldwide occurring in developing countries.^13^ Inflammation may play an important role in the progression of stroke, and inflammatory molecules might become both biomarkers and therapeutic targets for stroke management.^14^ The infection markers and inflammatory molecules, such as CRP, WBC, and IL‐6 were suggested to be biomarkers for outcome of ischemic stroke.^15^, ^16^, ^17^ Compared with these markers, PCT was not a widely diagnostic marker, it is considered to be the best available biomarkers to diagnose infection.^18^ In a recent study, PCT was suggested to be an independent risk factor for ischemic stroke.^19^ However, the prognostic value of serum PCT in acute ischemic stroke was little to know.^10^

In his study, our results showed that the concentrations of PCT in patients who died in 30 days were significantly higher than those who survived. As shown by multivariate logistic regression PCT level was an independent prognostic marker of 30‐day mortality. And Kaplan‐Meier analysis indicated PCT levels might help to predict 30‐day mortality. These results suggested that the concentration of PCT strongly predicted all‐course mortality in patients with acute ischemic stroke in first 30 days. However, hypertension and coronary artery disease, which could increase the incidence of ischemic stroke,^20^, ^21^ were not associated with the 30‐day mortality of ischemic stroke in our data. Moreover, in our data, PCT levels were also significantly higher in patients with unfavorable functional outcome compared with those in patients with a favorable outcome, which was consistent with previous report.^22^

Procalcitonin was a specific inflammatory biomarker, and systematic inflammation plays a crucial role in the pathogenesis of ischemic stroke. Thus, the increased PCT level in serum may due to the inflammatory process in acute ischemic stroke. The increased level of PCT was also observed after severe trauma,^11^ so we suspected that the increased level of PCT in acute ischemic stroke may also partly because the tissue injury after stroke. In this study, we found that PCT level was associated with the 30‐day mortality in Chinese patients, this may indicate that PCT was an independent prognostic marker of short‐term mortality after one set of ischemic strokes.
